# An Elevated Neutrophil-to-Lymphocyte Ratio Predicts Poor Prognosis in Patients with Liver Cancer after Interventional Treatments

**DOI:** 10.1155/2022/6141317

**Published:** 2022-11-25

**Authors:** Xiaonan Li, Yongsheng Zhang, Wei Ma, Jingwen Li

**Affiliations:** ^1^Department of General Practice, Shandong Provincial Hospital Affiliated to Shandong First Medical University, Jinan, China; ^2^Department of Gastroenterology, Shandong Provincial Hospital Affiliated to Shandong First Medical University, Jinan, China

## Abstract

This study is aimed at examining the prognostic value of blood neutrophil-to-lymphocyte ratio (NLR) in patients with hepatocellular carcinoma (HCC). Demographic and clinical data of 543 HCC patients treated with interventional therapies were retrospectively analyzed. Preoperative NLRs were determined and receiver operating characteristic (ROC) curves were plotted for survival time in patients with high (NLR ≥3.8) and low (NLR<3.8) NLR. The median overall survival (OS) was 1241 days after interventional therapies and was significantly reduced in the high NLR group when compared to the low NLR group. The median progression-free survival time (PFST) of patients was also significantly shorter in the high NLR group than in the low NLR group. Univariate analysis revealed that tumor type, therapy method, maximum tumor size (>3 mm), and NLR (>3.8) were risk factors for OST and PFST (*P* < 0.05). Multivariate analysis indicated that tumor type, maximum tumor diameter, therapy method, and NLR (>3.8) were independent risk factors for PFST (*P* < 0.05). Our results demonstrate that preoperative NLR has prognostic value for patients with HCC undergoing interventional therapies, and high NLR is an indication of poor prognosis.

## 1. Introduction

Liver cancer is one of the leading malignant tumors in the world and ranks the fourth in the causes of cancer-related death [1, 2]. For patients diagnosed with hepatocellular carcinoma (HCC) of all stages, the overall 5-year survival rate is estimated to be about 18%, and the incidence is increasing year by year. For instance, the incidence is about 18.3 per 1,000,000 persons in China, and the mortality rate is about 17.1/100000 [3]. Orthotopic liver transplantation (OLT) is one of the best treatment options for liver cirrhosis and HCC. However, due to insidious onset of HCC, a majority of patients are already at late stage once diagnosed, and only less than 20% can be treated with OLT or surgically [4, 5]. It is therefore important to develop prognostic biomarker to better manage patients for this disease.

Inflammation-related prognostic indicators have been related to the survival and other prognostic parameters such as tumor aggressiveness [6]. They include a number of easily measurable indicators of inflammation that can be obtained in routine clinical blood-based tests, such as counts and levels of neutrophils, lymphocytes, monocytes, platelets, albumin, C-reactive protein (CRP), and monocyte-to-lymphocyte ratios, among others [7–9]. Neutrophils in human peripheral blood have the functions of phagocytosis, chemotaxis, and bactericide, and lymphocytes are involved in the immune response [10, 11]. Studies have shown that the normal NLR values in an adult, nongeriatric, population in good health are between 0.78 and 3.53 [12], neutrophil-to-lymphocyte ratio (NLR) has a potential as prognostic marker, and the elevated NLR is associated with poor prognosis of diseases and cancers such as breast cancer [13], gastric cancer [14, 15], advanced melanoma treated with nivolumab [16], pancreatic cancer [17], in extensive-stage small cell lung cancer [18], and others [19, 20]. However, it is unknown whether if it has prognostic value for HCC patients after interventional therapies.

In the present study, we retrospectively analyzed the relationship between the ratio and survival of HCC patients after interventional therapies.

## 2. Materials and Methods

### 2.1. Patients

This is a single-center retrospective study. The medical records of patients who underwent interventional therapies for HCC between January 1, 2015 and December 31, 2019 at Shandong Provincial Hospital Affiliated to Shandong First Medical University, Jinan, China, were retrieved and analyzed. Patients were included if they fulfilled the following inclusion criteria: (1) had complete baseline clinical data for HCC, including CT scan findings on HCC size, (2) histologically proven HCC, and (3) received hepatectomy and other treatments for HCC. Patients were excluded if (1) distant metastasis was found at the first visit, (2) treated for other cancers within 6 weeks, (3) had severe infections or any hematology-related diseases, and (4) administered with any immunosuppressive medications within 6 months. Data retrieved from the hospital electronical medical data system included age, gender, history of smoking, maximum tumor diameter (MTD), pathological type, therapeutic method, and distant metastasis. This work was reported in line with the STROCSS criteria [21] and was approved by the Research Ethic Committee of Shandong Provincial Hospital Affiliated to Shandong First Medical University, Jinan, China (approval number: SPH-CR-2316, July 2020). Written informed consent was obtained from every patient.

### 2.2. Data Collection

Peripheral venous blood samples (10 ml) were drawn from all patients within 2 days prior to the interventional therapies and used to assess neutrophil and lymphocyte counts. NLR was calculated and used to plot receiver operating characteristic (ROC) curve for postoperative median survival time. Since NLR was 3.8 at the maximum Youden's index, this value was used as optimal cut-point to group the patients into high and low NLR groups. The Youden index is a main summary statistic that measures the potential effectiveness of a biomarker based on the ROC curve [22]. The cut-point that achieves this maximum is referred to as the optimal cut-point because it is the cut-point that optimizes the biomarker's differentiating ability when equal weight is given to sensitivity and specificity [23, 24].

### 2.3. Follow-Up and Survive Survey

After therapy, patients were followed-up regularly via phone and mail till June 31, 2020. The follow-up lasted up to 1820 day, with a medium follow-up time of 1113 days. Overall survival (OS) time and progression-free survival (PFS) time were calculated.

### 2.4. Statistical Analysis

The Student *t*-test was used to compare normally distributed data. A chi-square test was used for categorical variables. Receiver operating characteristic (ROC) curves were constructed, and the areas under the curves (AUCs) were calculated to evaluate the predictive abilities of the NLR for discriminating patients with good and poor prognosis. Overall, progression-free survival rate of patients was estimated through Kaplan-Meier survival analysis. The log-rank test was used to compare survival rates for groups for high and low NLR. Prognostic factors were assessed using univariate and multivariate analyses (Cox proportional hazard regression model). Data were analyzed using IBM SPSS Statistics (v. 20.0, IBM, New York, USA). *P* < 0.05 was considered statistically significant.

## 3. Results

### 3.1. Baseline Characteristics

A total of 599 patients were found satisficing the inclusion criteria. Among them, 56 patients were later excluded due to various reasons, including the loss of follow-up, and the clinical data for the remaining 543 patients were collected and analyzed. The study population consisted of 287 males and 256 females with a median age of 55.8 years (range 32-82 years).

**Figure 1:**
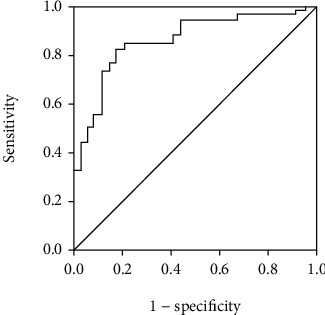
The ROC curve of neutrophil-to-lymphocyte ratio for predicting overall survival time in patients with HCC after interventional therapy.

Among them, 151 were smokers, and most cancers had not distant metastasis. The MTD ranged from 3 to 7 mm. ROC curve for postoperative OS time revealed that NLR was 3.8 at the Youden index, and AUC was 0.891. For the prediction at this point, the sensitivity was 84.4% and specificity was 86.5% (Figure 1). This value was used to group the patients into high (≥3.8, *n* = 256) and low (<3.8, *n* = 287) NLR groups. Analysis showed that the two groups were not statistically different in age, gender, history of smoking, MTD, tumor site and type, and therapeutic methods but their NLR values were different (3.8-5.4 in high vs 2.1-3.8 in low NLR groups (Table 1).

### 3.2. High NLR Reduces Survival Time

**Figure 2:**
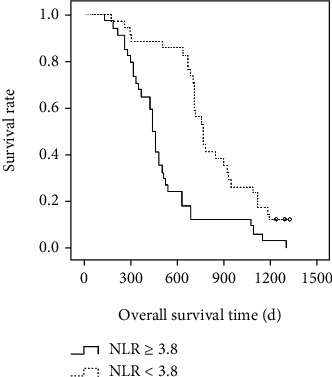
The Kaplan*-*Meier curves for overall survival times in patients with HCC and different neutrophil-to-lymphocyte ratios (NLR) after interventional therapy.

**Figure 3:**
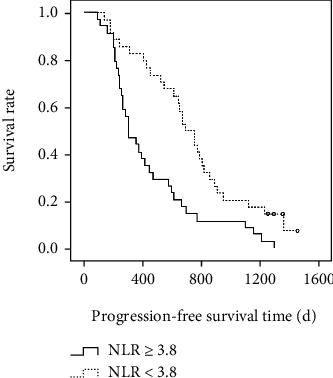
The Kaplan*-*Meier curves for progression-free survival time in patients with HCC and different neutrophil-to-lymphocyte ratios (NLR) after interventional therapy.

After the interventional therapy, patients were followed-up for up to five years. By the end of this study, 309 patients died and 234 were alive. Taken all patients together, the median OS time was 1241 days, and 1- and 2-year OS rates were 64.10% and 32.80%, respectively. For patients with high NLR (≥3.8), the median OS time was 381 days, and 1- and 2-year OS rates were 33.10% and 12.30%, respectively, and for patients with low NLR (<3.8), the median OS time was 1465 days and 1- and 2-year OS rates were 85.40% and 43.60%, respectively. The difference in the survival time and rates were statistically significant between the high and low NLR groups (*P* < 0.01, Figure 2). The PFS time was 529 days in all patients. However, the PFS time was significantly shorter in high than in low NLR patients, (242 vs 761 days, *P* < 0.05, Figure 3).

### 3.3. Factors Affecting Prognosis of OS and PFS

To analyze factors that affect OS and PFS after the therapy, we first performed univariate analysis, and the results indicated that the tumor type, therapy method, MTD, and NLR were significantly related to postoperative OS time and PFS time (*P* < 0.05, Tables 2 and 3). On other hand, other demographic and clinical features such as age, gender, smoking status, and tumor site were not significantly associated with the OS time and PFS time. These significantly related variables were then included in multivariate regression models for further analysis. The results revealed that therapy method, MTD, and NLR were the independent risk factors affecting OS time and PFS time (*P* < 0.05, Table 4).

## 4. Discussion

Most HCC patients are in the middle and late stages when diagnosed and may have missed the optimal surgery time [25]. Several treatment options are available for HCC patients, and among them, OLT and surgical resection are the mainstay treatments, although personalized therapies such as transarterial chemoembolization (TACE), transarterial radioembolization (TARE), and stereotactic body radiation (SBRT) as well as immunotherapy for HCC are being developed to improve overall survival [4, 26]. However, the overall survival of patients is still not satisfactory after the therapeutic processes due to various reasons. Therefore, discovery of indicators that can predict the prognosis for HCC patients is highly demanded. Studies have shown that the occurrence and progression of HCC is related to inflammation over a long period [27, 28]. NLR is an indicator of inflammation and is shown to be associated with prognosis of a variety of tumors [29]. Increased number of neutrophils in tumor and deceased lymphocyte count often indicate poor prognosis in the cancer patients [30, 31] or patients after selective internal radiation therapy [32].

Since both lymphocytes and neutrophils mainly play roles in protecting the body from infections and are a part of the immune system, they are associated with prognosis of diseases, including cancers. For example, lymphocyte was shown to be able to predict the severity and prognosis in patients with HBV-related acute-on-chronic liver failure [33] and lung cancer [34]. Neutrophils increase as a result of detrimental outcome in several tumors [35]. However, in our patient data, no relationship between either absolute neutrophil or leucocyte count alone was found to be significantly associated with the prognosis.

NLR has been reported as a potential prognostic marker, and the elevated ratio is associated with poor prognosis of different cancers such as breast cancer [13], gastric cancer [14, 15], advanced melanoma treated with nivolumab [16], pancreatic cancer [17], and in extensive-stage small cell lung cancer [18]. However, due to the heterogenicity of patient populations, the relationship needs to be analyzed for different patient populations to develop a cut-off that is best fit for specific patient groups (or at least large patient groups), because neutrophil and lymphocyte may vary in response to many factors, including treatment protocols, drugs, and methods. For HCC, NLR was found to accurately predict the probability of long survival after sorafenib treatment [36], and increased NLR was associated with poor survival after selective internal radiation therapy [32]. However, NLR variation after surgical section in HCC has not been well addressed.

In this study, we focused on HCC patients mainly after surgical sections and other interventional therapies such as liver transplant and radiation therapy and found that the NLR are related to OS and PFS times, and high NLR predicts shorter OS time and PFS time and has significant prognostic value. This is consistent with the previous results in lung cancer [37–39]. In addition, NLR is found to be related to the recurrence, metastasis, and prognosis of a number of solid tumors, such as esophageal cancer [40], prostatic cancer [41], and cervical cancer [42], and liver cancer [43] is useful in analyzing allergic conditions, inflammatory disorders, and infectious diseases [44, 45]. Different from other clinical indicators such as tumor size and grading, which require use of relatively complex and invasive surgical procedure, neutrophil and lymphocyte counts are readily available in routine blood tests. Therefore, NLR is a convenient biomarker for predicting the prognosis of HCC patient and can be used to stratify patients before different surgical and interventional treatment options. For instance, patients with high NLR could be allocated to receive relatively less invasive surgery to reduce their postoperative risk. On other hand, patients with low NLR may be tolerant to liver transplant and section. In addition, NLR could be monitored over the therapeutic periods as an auxiliary index for the progress and outcome of HCC after treatment. However, since the lymphocyte and neutrophil counts are affected by many factors, especially infections [46, 47] and drugs [48], and in a recent study, COVID-19 infection was also found to result in severe lymphopenia [49], therefore, cautions should be taken to interpret the changes of NLR in HCC patients, and additional data, particularly inflammation-related data, are needed to trace the therapeutic outcomes and to rule out other factors and diseases that may affect changes. For example, the pathogenesis of several diseases such as cardiovascular diseases [50], retinal artery occlusion [51], and spinal epidural abscess [52] have been found to result in high NLR, while treatment with 25-hydroxyvitamin D 3 and smoking cessation are associated with a reduced blood NLR [53, 54], suggesting when NLR is used for individual patients, it should be evaluated along with other pathological conditions to obtain more reliable prediction.

Mechanisms by which high NLR are associated with poor HCC prognosis may result from the interaction between tumor and inflammatory microenvironment [55, 56]. Immune cells such as activated macrophages, stellate, and mast cells have the ability to infiltrate into tumors, leading to increased tumor growth [57]. The peritumor infiltration by neutrophils may trigger inflammatory response to release free radicals and angiogenic response to enhance tumor growth [58, 59]. In addition, therapy method and MTD were also found to be related to the survival of HCC patients after treatments. This is consistent with early studies [38, 60, 61].

There are limitations in this study. This study is a single-center retrospective analysis; the sample size is relatively small. However, it may serve as starting point for multicenter and large prospective study in the future to further validate our conclusions for HCC patients.

Taken together, our study demonstrated that blood NLR may be used as prognostic marker to predict the prognosis of HCC for patients with middle and later stage HCC after interventional therapy. The preoperative NLR values may be used to stratify patients for different surgical and interventional treatment options before treatments and to monitor postoperatively the progress and outcomes of treatments.

## Figures and Tables

**Table 1:** Comparison of baseline date of HCC patients in high and low NLR groups.

Variables	*NLR* ≥ 3.8 (*n* = 256)	*NLR* < 3.8 (*n* = 287)	*X* ^2^	*P*
Age (year)			0.211	0.242
≥60	126	147		
<60	130	140		
Gender			0.357	0.117
Male	135	152		
Female	121	135		
Smoking			1.103	0.160
Yes	66	85		
No	190	202		
Tumor location			0.930	0.167
Upper liver	121	138		
Lower liver	135	149		
Tumor type			0.514	0.265
HCC	233	263		
Cholangiocarcinoma	12	13		
Liver angiosarcoma	4	5		
Hepatoblastoma	7	6		
Therapy			3.476	0.076
Hepatectomy	165	180		
Liver transplant	35	41		
Ablation	23	26		
Radiation therapy	21	18		
Chemotherapy	12	22		
Maximum tumor diameter			0.228	0.820
≥4 mm	145	155		
<4 mm	111	132		
NLR range	3.8-5.4	2.1-3.8	1.228	0.022

**Table 2:** Univariate analysis of factors affecting overall survival time.

Variables	*n*	Median OS time (day)	95% confidence interval	*P*
Age (year)				0.136
≥60	273	1186	815.3–1328.7	
<60	270	1386	862.8–1453.1	
Gender				0.423
Male	256	1286	915.2–1458.7	
Female	287	1387	962.8–1613.5	
Smoking				0.166
Yes	151	1186	1115.2–1498.7	
No	392	1287	1162.8–1576.5	
Tumor location				0.981
Upper liver	259	1286	1085.2–1518.7	
Lower liver	284	1327	1122.8–1586.5	
Tumor type				0.033
HCC	496	1286	1004.1–1675.8	
Cholangiocarcinoma	26	1331	900.7–1773.2	
Liver angiosarcoma	9	931	570.7–1273.2	
Hepatoblastoma	13	523	334.9–1155.0	
Therapy method				0.027
Hepatectomy	345	1226	934.9–1545.0	
Liver transplant	76	1321	907.7–1523.2	
Ablation	49	923	534.9–1155.0	
Radiation therapy	39	977	524.9–1295.0	
Chemotherapy	34	1277	914.9–1405.0	
Maximum tumor diameter				0.05
≥4 mm	300	1177	801.4–1342.5	
<4 mm	243	1477	1101.4–1642.5	
NLR				0.000
≥ 3.8	256	381	218.8–535.1	
<3.8	287	1465	1028.8–1605.1	

**Table 3:** Univariate analysis of factors affecting progression-free survival time.

Variables	*n*	Median OS time (day)	95% confidence interval	*P*
Age (year)				0.116
≥60	273	516	215.3–628.7	
<60	270	566	262.8–723.1	
Gender				0.471
Male	256	526	115.3–718.7	
Female	287	576	222.8–893.1	
Smoking				0.176
Yes	151	486	115.2–598.7	
No	392	687	161.8–826.5	
Tumor location				0.955
Upper liver	259	586	285.2–718.7	
Lower liver	284	627	322.8–986.5	
Tumor type				0.023
HCC	496	476	104.1–575.8	
Cholangiocarcinoma	26	331	90.7–473.2	
Liver angiosarcoma	9	231	70.7–373.2	
Hepatoblastoma	13	123	74.9–265.0	
Therapy method				0.027
Hepatectomy	345	528	134.9–545.0	
Liver transplant	76	321	107.7–623.2	
Ablation	49	223	84.9–455.0	
Radiation therapy	39	577	124.9–795.0	
Chemotherapy	34	477	114.9–605.0	
Maximum tumor diameter				0.021
≥4 mm	300	177	81.4–342.5	
<4 mm	243	777	101.4–942.5	
NLR				0.000
≥ 3.8	256	242	98.8–425.1	
<3.8	287	761	428.8–905.1	

**Table 4:** Multivariate analysis of factors affecting overall survival time and progression-free survival time.

Variable	HR	*P*	95% CI
Overall survival time			
Therapy method	0.68	0.025	0.30-1.01
MTD	4.18	0.011	2.21-7.45
NLR ≥3.8	5.79	0.013	3.62-7.76
Progression-free survival time			
Therapy method	0.50	0.035	0.32-1.02
MTD	4.28	0.021	2.02-8.12
NLR ≥3.8	3.59	0.011	2.16-4.19

## Data Availability

The datasets used during the current study are available from the corresponding author on reasonable request.
